# Non-obstetric surgery during pregnancy – an eleven-year retrospective analysis

**DOI:** 10.1186/s12884-019-2554-6

**Published:** 2019-10-25

**Authors:** J. Vujic, K. Marsoner, A. H. Lipp-Pump, P. Klaritsch, H. J. Mischinger, P. Kornprat

**Affiliations:** 1 Department of General Surgery, Medical University of Graz, Auenbruggerplatz 29, A-8036 Graz, Austria; 2 Department of Gynecology and Obstetrics, Medical University of Graz, Graz, Austria

**Keywords:** Nonobstetric surgery, Pregnancy, Appendicitis, Cholecystitis, Obstetrics

## Abstract

**Background:**

Diagnosis and management of non-obstetric abdominal pathologies during pregnancy are clinically challenging for both obstetricians and general surgeons. Our aim was to evaluate the outcome of pregnant patients who had undergone non-obstetric abdominal surgery.

**Methods:**

We retrospectively reviewed 76 pregnant patients who had required surgery for non-obstetric abdominal pathologies during pregnancy at our department from January 2005 to December 2015. Data were collected retrospectively from medical records as well as from our institutional perinatal database. We evaluated data for clinical presentation, perioperative management, preterm labor, and maternal and fetal outcomes.

**Results:**

The patients’ mean age was 29 (interquartile range IQR 25–33) years. Indications for surgery were acute appendicitis in 63%, adnexal pathology in 11%, cholecystolithiasis in 5% and other indications in 21%; surgery was performed in an elective setting in 18% and in an emergent/urgent setting in 82%. In five cases, complications, three of them oncological, called for further surgery. Ninety-seven percent of operations were conducted under general anesthesia. Median skin-to-skin time was 50 (37–80) minutes, median in-hospital stay was 4 (3.5–6) days, and 5 % required postoperative intensive care. Preterm labor occurred in 15%, miscarriage in 7% (none of them directly related to abdominal surgery).

**Conclusion:**

Abdominal surgery for non-obstetric pathology during pregnancy can be performed safely, if mandatory, without increases in maternal and fetal pathology, miscarriage, and preterm birth rates.

## Background

Diagnosis and management of non-obstetric abdominal pathologies during pregnancy are clinically challenging for both obstetricians and general surgeons. The number of patients requiring surgical intervention for non-obstetric indications ranges from 0.75–4.8% [1–3] in retrospective series. Obviously, all published data on non-obstetric surgery in the pregnant patient originate from retrospective case series and a few meta-analyses; no randomized trials are available for this special patient collective and its optimal clinical management. In 2016, Norwitz and coworkers proposed guidelines for the optimized management of the pregnant patient undergoing non-obstetric surgery based on a recent review of observational studies, extrapolation from trials during cesarean delivery, and expert opinions [4].

The most frequent indications for surgery during pregnancy are infections such as acute appendicitis and cholecystitis [5–14]; pregnant women may also require acute surgical intervention for ovarian disorders and bowel obstruction, as well as for traumatological or oncological indications [11]. Any kind of pathology can occur in pregnant women and require immediate surgical treatment and optimized interdisciplinary management to achieve maximum safety for both mother and fetus, to avoid teratogenous medication, fetal acidosis and hypoxemia, and adverse pregnancy outcomes such as miscarriage, stillbirth or premature birth [4, 8].

There are no clear recommendations as to the circumstances in which laparoscopy should be used in pregnant women or open surgery preferred; this decision is mostly taken at the surgeon’s discretion. Only for unclear acute abdomen with suspected acute appendicitis does the Society of American Gastrointestinal Endoscopic Surgeons recommend laparoscopy [12, 13].

We aimed to evaluate the outcome of pregnant patients undergoing non-obstetric abdominal surgery at our department.

## Methods

Our patient collective was a reference group of 76 pregnant patients who underwent surgery for non-obstetric abdominal pathologies at our department from January 2005 to December 2015. Data were collected retrospectively from medical records as well as from our institutional perinatal database. Surgical technique: Elective surgeries were delayed to the second trimester, whenever possible. If there was an elective indication for surgery during the second or third trimester, surgery was postponed until after delivery; in selected cases, non-obstetric surgery was performed during elective cesarean section. Acute surgical interventions, mostly for infectious surgical indications such as appendicitis and cholecystitis, were performed irrespective of gestational age.

Anesthesia was performed according to standard procedures for adult patients; the fetal heart rate was always documented pre- and postoperatively. When feasible in the operative setting, the fetal heart rate was monitored continuously after the 24th week of pregnancy. Intraoperative tocolysis was administered intravenously in 17 Patients via continuous perioperative infusion with Hexoprenalin (Gynipral®) and in some cases Atosiban (Tractocile®) after the 24th pregnancy week. The Atosiban (Tractocile®) is in clinical usage since February 2000, and was used in our study in four cases. In other 12 cases tocolysis was performed with Hexoprenalin (Gynipral®).

In advanced pregnancy, non-obstetric surgery was performed in the gynecological operating theater with a caesarian section team on standby.

## Results

The mean age of the 76 patients included in the retrospective analysis of the 11-year period was 29 (interquartile range IQR 25–33) years (Table 1). The predominant indication for non-obstetric surgery was acute appendicitis in 63%, adnexal pathology in 11%, cholecystolithiasis in 5%, and other indications in 21%. Surgical interventions were elective in 18% (14 patients) of the cases and emergent in 82% (62 patients). Surgical reintervention was necessary in five (7%) cases to secure a successful result, and in three cases for oncological reasons. Ninety-seven percent of operations were conducted under general anesthesia. Depending on the type of surgery and individual case, preoperative prophylactic antibiotics were administered to 44 (56%) of our patients, while 16 (22%) of the patients required therapeutic antibiotics (Table 1). Glucocorticoids were administered 24–48 h before the surgery in only 6 of the cases as support for fetal lung maturation only if the intervention occurred between the 24th and 34th weeks of pregnancy, and only in the absence of systemic infection. Median skin-to-skin time was 50 (37–80) minutes. Intraoperative tocolysis was administered in 17 patients (22%). The median hospital stay at our department of general surgery was 4.5 days (4–6). Five percent of patients required postoperative intensive care (Table 2). The gestational age during which most (58%) of the non-obstetric surgical interventions were performed was the second trimester; 17 of our surgical interventions (22%) were in the third trimester (Table 3).

**Table 1:** General study data

Study data	n (%), overall: *n* = 76/ median (IQR)
Age (Years)	29 (25–33)
OP time (min)	50 (37–80)
Appendectomy	48 (63%)
Cholecystectomy	4 (5%)
Other surgical interventions	16 (21%)
Ovarian surgery	8 (11%)
Elective surgery	14 (18%)
Urgent surgery	44 (58%)
Emergency surgery	18 (24%)
Laparoscopic surgery	19 (25%)
Preoperative antibiotics	44 (56%)
Therapeutic antibiotics	16 (22%)

**Table 2:** Intra- and postoperative data

Intraoperative data	N (%), overall *n* = 76
Intervention in general anesthesia	74 (97%)
Local/spinal anesthesia	4 (5%)^a^ Conversion to general anesthesia in 2 cases
Intraoperative tocolysis	17 (22%)
Postoperative maternal complications	4 (5%)
Postoperative fetal complications	5 (7%)
Postoperative ICU stay (days)	4 (5%)
Surgical reintervention	5 (7%)
Preoperative glucocorticoids	6 (8%)
OP 1st trimester	15 (20%)
OP 2nd trimester	44 (58%)
OP 3rd trimester	17 (22%)
Gestational age (weeks)	18.5 (14–25)
Hospital stay (days)	4 (3.5–6)

* conversion to general anesthesia in 2 cases

**Table 3:** Follow-up with pregnancy outcome details

Outcome details	N (%), median (IQR)
Miscarriage	5 (7%)
Gestational age at delivery	40 (37–41)
Premature birth	12 (16%)
Spontaneous birth	24 (32%)
Vaginal-surgical	4 (5%)
Secondary Caesarean	13 (17%)
Primary Caesarean	18 (24%)
Caesarean in non-obstetrical department	7 (9%)
Neonatal morbidity	9 (12%)
Neonatal mortality	0
Maternal mortality	0

After follow-up of patients who had non-obstetric surgical interventions during pregnancy, it was noted that the median pregnancy week reached after the intervention was 40 (37–41). The details of the follow-up are shown in Table 3. Preterm labor occurred in 12 cases (16%), and miscarriage in 5 cases (7%), with none of them directly related to abdominal surgery. In fact, the follow-up documented no case of neonatal mortality, and 9 (12%) cases of neonatal morbidity. Spontaneous birth was reported as most frequent, with 24 cases (32%). Caesarian section was primary in 18 (24%) cases, and secondary in 13 (17%) cases. Caesarian delivery was performed in the department of surgery in 7 (9%) patients.

As acute appendicitis is the most common non-obstetrical surgical intervention during pregnancy, special attention was paid to the detailed data of this predominant group of patients (48 of 76 cases). The detailed data on the appendectomy cohort is presented in Table 4. The prevalent operational approach was open appendectomy in 42 cases, while a laparoscopic approach was used in only 6 cases. The two-sided *p* value showed that there were no consequences for gestational week at birth (mean value 4 and 39). Antibiotic therapy was administered only in open appendectomy in 11 cases (28%). Glucocorticoids were also administered only in open appendectomy in 2 cases (5%). Of the patients who underwent open appendectomy, two showed perioperative morbidity (5%) and three had three miscarriages (7%). There was no statistically significant difference in lengths of hospital stay for patients who had open or laparoscopic appendectomy (mean value 4 and 5.5) (Table 4).

**Table 4:** Appendectomy data

Variable	Total cohort appendectomy (*n* = 48), n (%), median (IQR)	Open AE (*n* = 42)	Lap. AE (*n* = 6)	Two-sided *p*-value
Age (years)	29 (25–33)	29 (25–33)	32 (27–35)	0.35
OP time (minutes)	42 (35–57)	42 (35–53)	50 (38–87)	0.32
Gestational week at surgery (weeks)	18 (12–21)	19 (15–23)	12 (10–18)	0.05
Gestational week at birth	40 (38–41)	40 (38–40)	39 (39–41)	0.47
Birth weight (g)	3330 (2918–3618)	3270 (2902–3560)	3400 (3214–3586)	0.12
Tocolysis	10 (21%)	10 (24%)	0	0.32
Antibiotic therapy	11 (24%)	11 (28%)	0	0.31
Glucocorticoids	2 (4%)	2 (5%)	0	1
Preterm birth^a^ (13/48 Birth not at the Clinic)	4 (8%)	4 (10%)	0	0.35
Maternal perioperative morbidity	2 (4%)	2 (5%)	0	1
Miscarriage	3 (6%)	3 (7%)	0	1
Hospital stay (days)	4.5 (4–6)	4 (3.75–5)	5.5 (4.5–6)	0.238

* 13/48 birth not at the clinic

## Discussion

Non-obstetric acute abdomen during pregnancy can be a diagnostic and therapeutic challenge. Pregnancy can obscure the clinical diagnosis; the perfomance of clinical examinations is still controversial [6], and the debate on the safest surgical approach during pregnancy continues.

The diagnosis of acute appendicitis is based mainly on the history and physical examination, which is less reliable in pregnant women, who undergo patho-physiological changes that favor relative immune suppression, thus altering the inflammatory response [4,5,9,14,15,16,]. The diagnosis of acute abdomen for non-obstetric complications (mostly appendicitis or cholecystitis) is performed using ultrasound and magnetic resonance imaging (MRI). Ultrasonography (USG) has a reported sensitivity of 67–100% and specificity of 83–96% for appendicitis in pregnancy. MRI is most meaningful in identification of a non-pathological appendix, thereby ruling out inflammation [15]. According to American College of Radiology, it is less reliable in detecting the presence of extraluminal air in perforated intestine, and therefore placed as second line imaging diagnostic method in this case [15]. In accordance with published studies, in our patient collective, acute appendicitis was the predominant reason for nonobstetric abdominal surgery during pregnancy [6, 14]. Complicated forms of acute appendicitis are reported to be more frequent in pregnant women and are associated with obstetrical complications such as premature delivery [4–6]. In our total collective, there were 12 premature births (16%), of which only 4 (10%) occurred after open appendectomy (none of which was characterized as complicated). Gök et al. suggested in their study that there is no significant difference in fetal or maternal outcome after open appendectomy and laparoscopic appendectomy [16]. The results of our study confirm their claim that both methods are safe, and that neither method led to fetal or maternal mortality [10]. Follow-up revealed no complications for either approach that were directly related to a negative pregnancy outcome.

The second most common reason for surgical intervention during pregnancy in our study was adnexal pathology, including one case of pediculated leiomyoma, the third was cholecystolithiasis [17–25].

Symptomatic gallstone disease in pregnancy has been reported to be related to increased mortality risk for both the mother and fetus [17, 20]. Cholecystolithiasis was diagnosed by using ultrasound (USG). USG is the diagnostic method of choice with a sensitivity of more than 95% [15]. Authors such as Sachs et al. stratified the risks of undergoing appendectomy and cholecystectomy in pregnancy, and concluded that approximately 5% of women experienced adverse obstetrical outcomes after appendectomy or cholecystectomy during pregnancy [2]. They found that cervical incompetence, sepsis, and other pre-existing states influenced adverse pregnancy outcomes, but not the surgical approach itself. These findings are in line with our results showing that surgical intervention had no influence on pregnancy outcome. Barut et al. and others have concluded that although conservative management of acute cholecystitis was favored, early surgical intervention showed better results [7]. Cholecystectomy was performed in our study in 4 cases (5%), without adverse effects on pregnancy.

Problems related to general anesthesia for nonobstetric interventions during pregnancy could be prevented by avoiding potentially dangerous drugs and securing adequate uteroplacental perfusion [3]. Up to now, no anesthetic drug has been shown to be clearly dangerous to the human fetus [21]. The decision on proceeding with surgery should be made by multidisciplinary team involving anesthesiologists, obstetricians, surgeons and perinatologists [8]. In our study, 74 (97%) of surgical interventions were performed in general anesthesia, with 4 cases of local/spinal anesthesia, of which 2 were converted to general anesthesia. Postoperative maternal complications developed in 4 cases (5%), with a mean ICU stay of 4 days, indicating the safety of general anesthesia. The American College of Obstetricians and Gynecologists published guidelines that proposed imperative consultation with obstetricians prior to nonobstetric surgery and other invasive procedures (e.g., coronary angiography or colonoscopy) because obstetricians are uniquely qualified to discuss aspects of maternal physiology and anatomy that may affect intraoperative maternal and fetal well being [8].

The study of Fong et al. focused on surgical interventions during the third trimester of pregnancy, giving the general recommendation that they should be avoided whenever possible [20]. In our study, 22% (17 cases) of all interventions were made the in third trimester, and went without significant complications. Yu et al. reported that an unfavorable pregnancy outcome after non-obstetric surgical intervention during pregnancy is based predominantly on inflammatory reaction and scar formation after nonobstetric surgery and postoperative hemorrhaging, none of which we found our study [1]. We reported 6 cases in which preoperative glucocorticoids could control the inflammatory response; the remaining 70 cases (92%) received no drugs preoperatively that could influence the inflammatory response, but without any significant adverse effect on postoperative complications. Huang et al. suggested that pregnant patients undergoing abdominal surgery showed more adverse events, with a slightly elevated risk of in-hospital mortality after nonobstetric surgery, than did nonpregnant patients [18]. These findings suggest a need to revise the protocols for postoperative care. None of the premature labors or miscarriages in our study was directly connected to surgical intervention. There is general agreement that surgical intervention should be performed in pregnant women whenever indicated; to our knowledge, there is no published report recommending general avoidance of surgical intervention during pregnancy, except for some recommendations concerning interventions in the third trimester [11, 14]. Further research on non-obstetric surgical interventions in pregnant women is warranted to develop guidelines for optimal pre- and postoperative management in pregnant women.

## Conclusion

Our study indicates that abdominal surgery for non-obstetric pathology during pregnancy can be performed safely whenever indicated without adverse obstetric outcome for either mother or fetus. In our study intraoperative treatment had no adverse influence on maternal or fetal pregnancy outcome.

## Data Availability

The datasets generated and/or analyzed during the current study are not publicly available due to Patient Data Protection Law (§ 37 Datenschutz in der Krankenanstalt, Stmk. Krankenanstaltengesetz 2012, Fassung vom 29.03.2019) but are available from the corresponding author on reasonable request.

## Abbreviations

ICU: Intensive care unit
IQR: Interquartile range
MRI: Magnetic resonance imaging
USG: Ultrasonography
